# Spectral Predictability of Soil Organic Matter Depends on Its Humin Fraction Rather than Spectral Fusion

**DOI:** 10.3390/s25247616

**Published:** 2025-12-16

**Authors:** Zhi Zhang, Meihua Yang, Asim Biswas

**Affiliations:** 1 Department of Environmental Engineering, Yuzhang Normal University, Nanchang 330103, China; zhangzhi@yuznu.edu.cn; 2 Key Laboratory of Nanchang City for Green New Materials and Industrial Wastewater Treatment, Nanchang 330103, China; 3 School of Environmental Sciences, University of Guelph, Guelph, ON N1G 2W1, Canada

**Keywords:** SOM, Humin fraction, humic acid, fulvic acid, vis–NIR, MIR, spectral fusion, LASSO regression, soil carbon monitoring

## Abstract

Soil organic matter (SOM) governs critical soil functions, including carbon storage, nutrient cycling, and microbial activity; yet the specific fractions responsible for its spectral predictability remain poorly understood. This study addresses a fundamental research gap by comparing visible–near-infrared (vis–NIR), mid-infrared (MIR), and fused spectroscopy for predicting SOM and its components: humic acid (HA), fulvic acid (FA), and Humin. Using 93 soil samples from subtropical croplands in southeastern China, we employed partial least squares regression with full spectra and LASSO-selected wavelengths to build predictive models. Results demonstrated that both vis–NIR and MIR individually provided moderately strong predictive performance for SOM and Humin (R^2^ = 0.79–0.90, CCC = 0.85–0.93), while FA remained unpredictable (R^2^ < 0.24) due to weak, overlapping spectral features. The strong predictability of SOM was primarily attributed to the Humin fraction, which comprises approximately 50 percent of total SOM and exhibits abundant spectrally active functional groups. Contrary to expectations, spectral fusion did not improve predictions because both spectral regions already contained complementary information, and fusion introduced redundancy and scale imbalance rather than increasing effective dimensionality. This study establishes that accurate SOM estimation depends fundamentally on the predictability and abundance of the Humin fraction, providing new mechanistic insights for spectroscopic soil carbon monitoring and highlighting the need for component-specific modeling approaches in soil organic matter research.

## 1. Introduction

Soil organic matter (SOM) constitutes a fundamental component of terrestrial ecosystems, regulating soil fertility, carbon sequestration, nutrient retention, and structural stability. However, SOM is chemically heterogeneous, comprising distinct fractions with varying stability, reactivity, and environmental functions. These fractions can be operationally classified into humic acid (HA), fulvic acid (FA), and Humin, each exhibiting unique molecular characteristics and ecological roles [1]. Humic acid, characterized by higher molecular weight and aromatic content, contributes significantly to soil buffering capacity and heavy metal complexation. Fulvic acid, with smaller molecular weight and greater solubility, serves as a mobile carrier for carbon and nitrogen transport in soil solutions. Humin, the most recalcitrant fraction, remains strongly bound to mineral surfaces and represents a major long-term carbon sink [2]. This functional differentiation within SOM directly influences soil physicochemical processes, microbial dynamics, and the overall carbon balance of terrestrial ecosystems [3,4].

Traditional wet chemical methods for quantifying SOM and its fractions face substantial limitations that constrain their application in contemporary research. Spectroscopic techniques offer promising alternatives for SOM quantification by exploiting the distinct absorption and scattering properties of soil constituents across the visible–near-infrared (vis–NIR) and mid-infrared (MIR) regions. In vis–NIR spectroscopy, absorption features arise from electronic transitions and overtones of molecular vibrations associated with organic matter, clay minerals, iron oxides, and carbonates [5]. Numerous studies have demonstrated that vis–NIR can predict soil organic carbon (SOC) with sufficient accuracy for rapid field screening applications [6,7]. Recent work has extended this capability to humus fractions, with reports indicating satisfactory prediction of FA (R^2^ = 0.73), HA (R^2^ = 0.56) and Humin (R^2^ = 0.83) using vis–NIR combined with machine learning algorithms [8,9]. Mid-infrared spectroscopy, which captures fundamental molecular vibrations of functional groups, has shown robust performance in predicting SOC and its fractions across large spectral libraries and diverse soil types [10,11]. Comparative analyses suggest that MIR often outperforms other spectral ranges for multiple soil properties and can effectively reveal compositional differences among carbon fractions [12].

Multi-sensor spectral fusion has emerged as a strategy to overcome the limitations of single-domain spectroscopy by integrating complementary information from different spectral regions. The rationale underlying fusion approaches is that vis–NIR captures electronic transitions and overtone-related features, whereas MIR provides direct information on molecular vibrations and functional groups. By combining these complementary sensitivities, fusion methods theoretically offer more comprehensive representations of soil composition [5,13]. However, despite the conceptual appeal, empirical evidence regarding the efficacy of spectral fusion remains inconsistent and often contradictory. While some studies report substantial improvements in SOC prediction accuracy through vis–NIR–MIR fusion under controlled laboratory conditions [14,15], others observe limited benefits or even decreased accuracy in soil classification tasks, attributed to overfitting and increased model complexity [16]. Moreover, the majority of fusion studies have focused on bulk SOM or total organic carbon, with little attention to individual SOM fractions and the mechanisms governing their spectral behavior.

Despite these advances, several critical knowledge gaps persist. First, it remains unclear which specific SOM fraction predominantly governs the spectral predictability of total SOM. For instance, Humin has been hypothesized to display more stable and soil-type–independent spectral responses than other SOM fractions due to its strong mineral associations [17]. However, to our knowledge, direct empirical evidence demonstrating consistent spectral behavior that can be unequivocally attributed to Humin remains scarce. Second, the existing fusion literature has focused almost exclusively on bulk SOM or total organic carbon, with minimal consideration of individual SOM fractions such as Humin, humic acid, or fulvic acid. This lack of mechanistic understanding and fraction-level evaluation represents a critical knowledge gap and highlights the need for studies that explicitly test whether fusion genuinely enhances the predictability of distinct SOM components rather than merely increasing spectral dimensionality. Third, the underlying mechanisms that explain why fusion sometimes improves or degrades prediction accuracy are poorly understood. Addressing these gaps is essential for developing mechanistically informed spectroscopic methods for soil carbon monitoring and for identifying the structural and chemical determinants of SOM spectral signatures.

**Figure 1:**
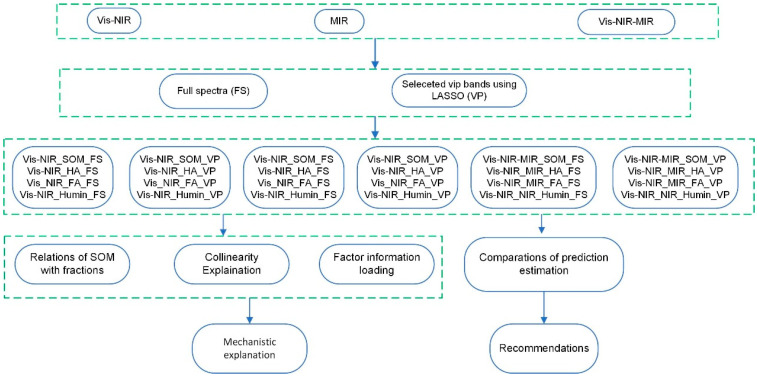
The research framework and analytical workflow.

This study was designed to address these research gaps through three specific objectives: (1) to compare the predictive performance of vis–NIR, MIR, and their fusion for SOM and its fractions (HA, FA, Humin) using both full spectra and variable-importance-based wavelength selection, (2) to identify which SOM component predominantly determines the overall predictability of SOM, and (3) to elucidate the mechanisms underlying changes in prediction accuracy resulting from vis–NIR–MIR spectral fusion. Figure 1 illustrates the overall research framework and analytical workflow employed in this investigation.

## 2. Materials and Methods

### 2.1. Study Area and Soil Sampling

**Figure 2:**
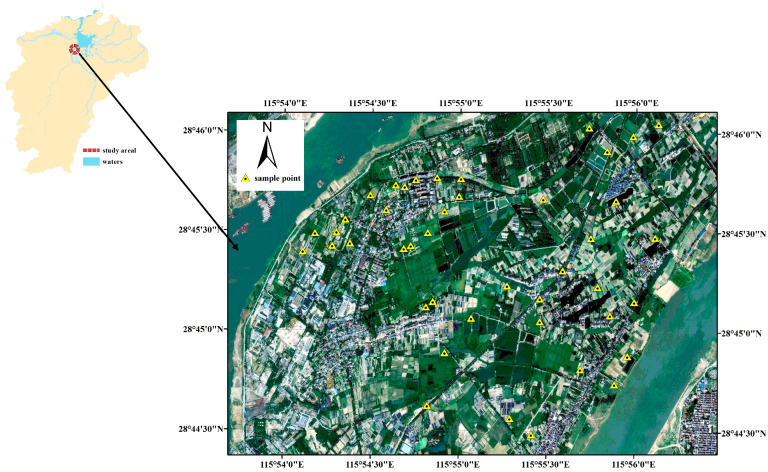
Location of the sampling area in southeastern China.

The study area is situated in the Yangzi region of Nanchang City, Jiangxi Province, southeastern China, within the Gan River floodplain of the middle Yangtze River Basin (Figure 2). The region is characterized by a flat terrain with minimal topographic relief, and soils are predominantly classified as Fluvisols, developed from Quaternary alluvial and fluvial deposits, with local occurrences of Cambisols and Gleysols on poorly drained areas, and Anthrosols formed under long-term intensive cultivation. In areas influenced by weathered red clay and sandstone materials, Acrisols/Alisols are also present. The key land use type from which samples were taken is cropland. The climate is humid subtropical monsoon, featuring hot and humid summers, mild winters, and abundant precipitation concentrated in spring and early summer. The study area represents a major peri-urban agricultural zone supplying a substantial proportion of Jiangxi Province’s fresh vegetable production, with intensive long-term cultivation history and high cropping intensity. These characteristics make the region an important representative site for investigating soil fertility dynamics, organic matter turnover, and sustainable soil management practices in the middle Yangtze River basin.

Soil sampling was conducted triennially beginning in 2019. Soil samples were collected following a stratified sampling design to capture local spatial variability. A total of 93 samples were taken from the topsoil (0–20 cm) across the study area. Each sampling point was georeferenced using a handheld GPS (±3 m accuracy), and three subsamples collected within a 2 m radius were composited to reduce small-scale microsite variability. Sampling locations were selected to represent contrasting land use types and soil conditions, thereby incorporating heterogeneity in texture, mineralogy, and organic matter content. All samples were air-dried at room temperature, ground to pass through a 2 mm sieve, and stored in sealed containers prior to chemical analysis and spectroscopic measurements.

### 2.2. Chemical Analysis

Soil organic matter content was determined using the potassium dichromate-sulfuric acid external heating method in accordance with Chinese agricultural standard NY/T 1121.6-2006 [18]. Organic carbon obtained by dichromate oxidation was converted to SOM using the conventional conversion factor of 1.724. Humus fractions, including HA, FA, and Humin, were determined through alkaline extraction and acid fractionation following Chinese forestry standard [19]. These operationally defined fractions represent chemically distinct pools with different environmental stabilities and functions.

### 2.3. Spectroscopic Measurement and Preprocessing

Visible–near-infrared spectra were acquired using a FieldSpec ProFR spectrometer (Analytical Spectral Devices, Boulder, CO, USA), covering the wavelength range of 350–2500 nm, with spectral resolutions of 3 nm (350–1000 nm) and 10 nm (1000–2500 nm). Before spectral acquisition, the instrument was allowed to warm up for 30 min to stabilize the detector. Prior to each measurement, the spectrometer was calibrated using a Spectralon panel with 99 percent reflectance. For each soil sample, thirty spectral measurements were collected at three random positions with ten internal replicates per position, and the average spectrum was used as the final representative spectrum. Spectra from wavelength regions 350–399 nm and 2451–2500 nm were excluded due to low signal-to-noise ratios, retaining the 400–2400 nm range for analysis.

Mid-infrared spectra were measured using an Agilent 4300 Handheld FTIR spectrometer (Agilent Technologies, Santa Clara, CA, USA) with a spectral range of 650–4000 cm^−1^. Sample preparation and measurement protocols were identical to those employed for vis–NIR spectroscopy to ensure consistency. Both vis–NIR reflectance (R) and MIR absorbance spectra were transformed using log(1/R) to approximate Beer–Lambert behavior and improve linearity with respect to analyte concentration. To reduce high-frequency instrumental noise, Savitzky–Golay smoothing with a second-order polynomial and an 11-point window was applied as the first preprocessing step. A first-derivative transformation (Savitzky–Golay, second-order polynomial) was then used to correct baseline shifts and enhance subtle absorption features relevant to SOM fractions. Spectra were subsequently normalized using standard normal variate (SNV) to minimize scattering effects associated with particle size and soil texture. Finally, noisy and low-signal regions at the edges of the detectors (e.g., 350–400 nm and >2450 nm in vis–NIR) were excluded. All spectra were resampled to uniform 10 nm intervals to facilitate spectral integration and fusion.

### 2.4. Predictive Modeling

Partial least squares regression (PLSR) was employed as the primary predictive modeling approach. PLSR is a widely used multivariate statistical method that projects both predictor variables (spectral reflectance) and response variables (soil properties) into a new latent space, identifying directions in the predictor space that maximize covariance with the response [20]. This approach is particularly well-suited for spectroscopic data characterized by high dimensionality, multicollinearity, and high noise-to-signal ratios. The optimal number of latent variables was determined via leave-one-out cross-validation, with the component corresponding to the lowest RMSECV selected as the final model.

To identify the most informative spectral wavelengths and reduce model complexity, the least absolute shrinkage and selection operator (LASSO) was applied for variable selection. LASSO introduces an L1 penalty term that shrinks regression coefficients and performs automatic feature selection by forcing less important coefficients to exactly zero [21,22]. The LASSO objective function is defined as follows:

$$minimize:\;{{\left||y\;-\;X\beta |\right|}_{2}}^{2}\;+\;\lambda {\left||\beta |\right|}_{1}$$

where y represents the target variable (SOM or its fractions), X denotes the spectral matrix, β is the coefficient vector, and λ is the regularization parameter controlling the degree of sparsity. LASSO hyperparameters were tuned using a log-spaced λ grid and 10-fold cross-validation with fixed random seeds to ensure reproducibility. The optimal λ was selected using the one-standard-error rule. All analyses were conducted using the glmnet package with version-controlled scripts. Selected wavelengths identified by LASSO were subsequently used to build PLSR models for comparison with full-spectrum approaches.

Spectral fusion was implemented using direct concatenation of vis–NIR and MIR spectra, creating a combined spectral matrix for model calibration. Both full-spectrum fusion and LASSO-based variable selection from the fused spectra were evaluated. This approach allows comparison of information gain from fusion relative to single-domain spectroscopy. In addition, advanced fusion approaches were not tested because our study aimed to establish a baseline assessment under limited sample size, where complex fusion methods would likely overfit and exceed the scope of this initial investigation (Table 1).

### 2.5. Spectral Multicollinearity Diagnosis

Singular value decomposition (SVD) was applied to the spectral matrix X to quantify spectral redundancy and evaluate multicollinearity. The SVD factorization is expressed as X = UΣV^T, where Σ is a diagonal matrix of singular values arranged in descending order. The condition number, defined as the ratio of the largest to smallest singular value (σ_1_/σ_n_), indicates the degree of multicollinearity, with values exceeding 30 suggesting strong redundancy and potential regression instability [20]. Cumulative singular value energy was calculated as the proportion of total spectral variance explained by the first k singular values [28], providing a quantitative measure of effective information dimensionality.

### 2.6. Model Evaluation Metrics

Model performance was assessed using multiple complementary metrics. The coefficient of determination (R^2^) quantifies the proportion of variance in the observed values explained by the model. Root mean square error (RMSE) and mean absolute error (MAE) provide measures of absolute prediction error in the same units as the target variable. The ratio of performance to interquartile range (RPIQ), calculated as (Q_3_ − Q_1_)/RMSE, offers a standardized performance metric less sensitive to extreme values than traditional RPD. Concordance correlation coefficient (CCC) evaluates both precision and accuracy by measuring agreement between observed and predicted values along the 1:1 line, combining correlation and bias components. Bias quantifies systematic over- or under-prediction. The Kennard–Stone (KS) algorithm was used to split the data into calibration (70 percent) and independent validation (30 percent) subsets to assess model generalizability.

All analyses were conducted in R (version 4.4.1) using the packages prospectr (v0.3.2), glmnet (v4.1-8), and pls (v2.8-2).

## 3. Results

### 3.1. Descriptive Statistics of SOM and Its Fractions

Descriptive statistics for SOM, HA, FA, and Humin are presented in Table 2. Soil organic matter content ranged from 13.29 to 67.02 g kg^−1^ in the calibration dataset and from 12.52 to 57.64 g kg^−1^ in the validation dataset, with means of 28.91 and 26.15 g kg^−1^, respectively. The complete range of validation samples fell within the calibration range, confirming appropriate dataset partitioning. Humic acid exhibited a similar distribution pattern, ranging from 4.77 to 28.23 g kg^−1^ with mean values of 12.07 and 10.93 g kg^−1^ for calibration and validation sets. Fulvic acid displayed the narrowest concentration range (1.64–6.51 g kg^−1^) and lowest mean values (3.17 and 2.96 g kg^−1^), reflecting its typically small and chemically unstable fraction within total SOM. Humin showed the widest absolute range (5.75–35.15 g kg^−1^) with mean values of 13.67 and 12.26 g kg^−1^, and accounted for approximately 40–69 percent of total SOM across all samples. Positive skewness values (2.04–2.71) indicate right-skewed distributions for SOM, HA, and Humin, suggesting that a subset of samples contained relatively high concentrations of these fractions.

### 3.2. Wavelength Selection Reveals Distinct Spectral Signatures

**Figure 3:**
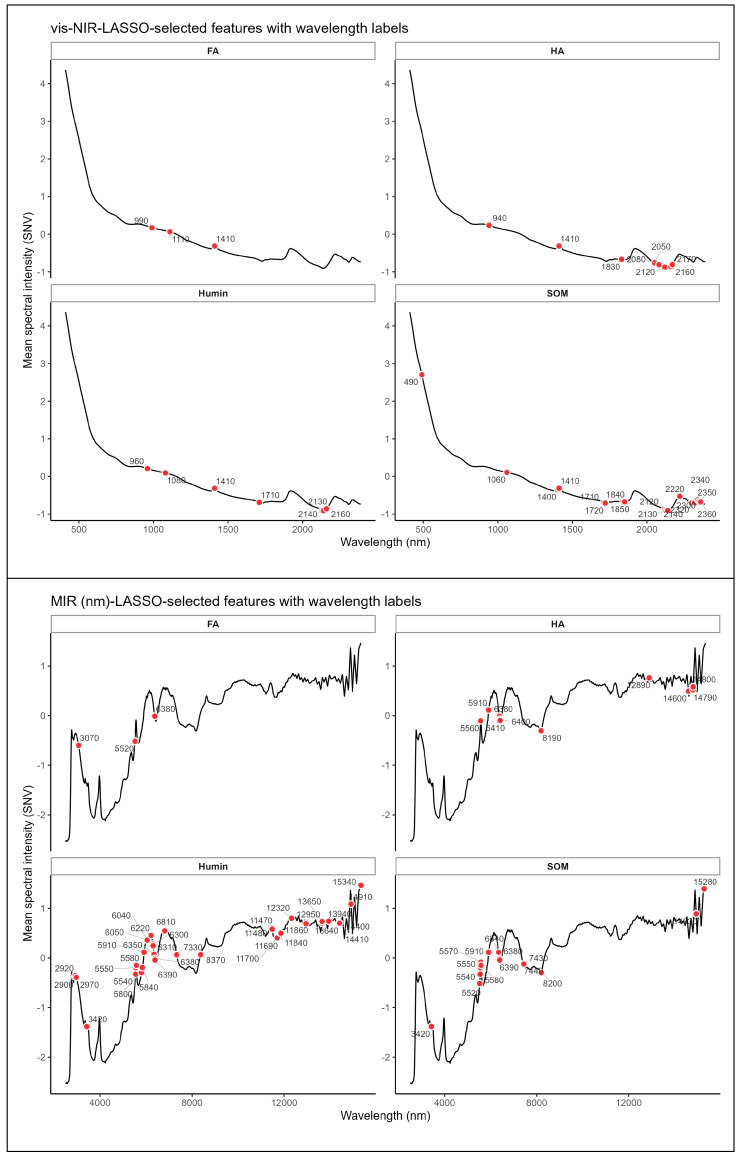
Variable importance wavelengths for SOM and its fractions selected using LASSO.

LASSO-based feature selection identified markedly different sets of informative wavelengths for SOM and its components (Figure 3). In vis–NIR spectroscopy, fulvic acid retained only three key wavelengths (990, 1190, and 1410 nm), reflecting its weak and diffuse spectral expression. Humic acid and Humin exhibited broader wavelength coverage, with approximately seven wavelengths (940–2160 nm) selected for HA and seven wavelengths (960–2160 nm) for Humin. Soil organic matter models retained the largest number of vis–NIR wavelengths (thirteen, spanning 490–2350 nm), demonstrating the broadest spectral sensitivity and reflecting its compositionally heterogeneous nature.

In MIR spectroscopy, fulvic acid selected four representative wavelengths (approximately 3070–13,800 nm), corresponding primarily to C–H stretching and O–H bending vibrational modes. Humic acid exhibited eight selected wavelengths (5560–14,790 nm), associated with aliphatic C–H stretching, aromatic C=C, and C=O functional groups. Humin identified the largest number of MIR wavelengths (more than twenty, spanning 2900–15,340 nm), reflecting its structurally complex and spectrally active composition rich in diverse functional groups. Soil organic matter models showed twelve selected wavelengths (3420–15,280 nm), distributed across regions characteristic of O–H, N–H, and Si–O combination modes. These distinct wavelength selection patterns underscore fundamental differences in the chemical composition and spectral behavior of SOM fractions.

### 3.3. Vis–NIR Prediction Accuracy for SOM and Its Fractions

**Figure 4:**
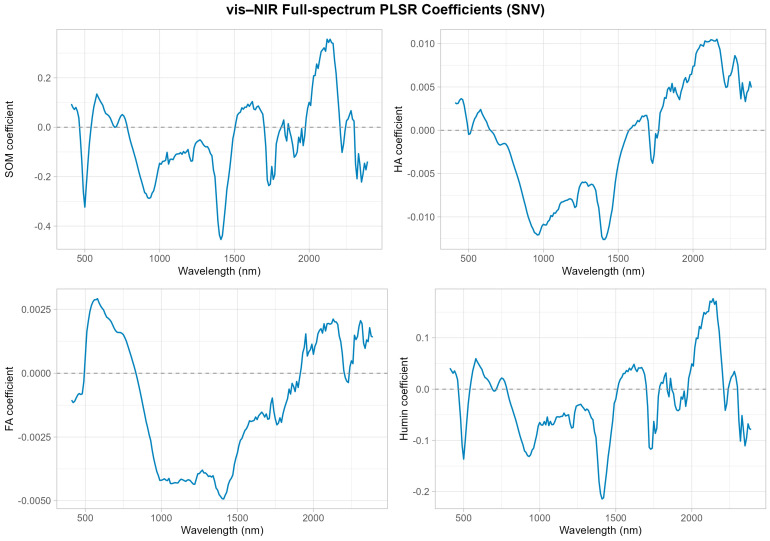
Regression coefficients for SOM and its fractions using full vis–NIR spectra.

**Figure 5:**
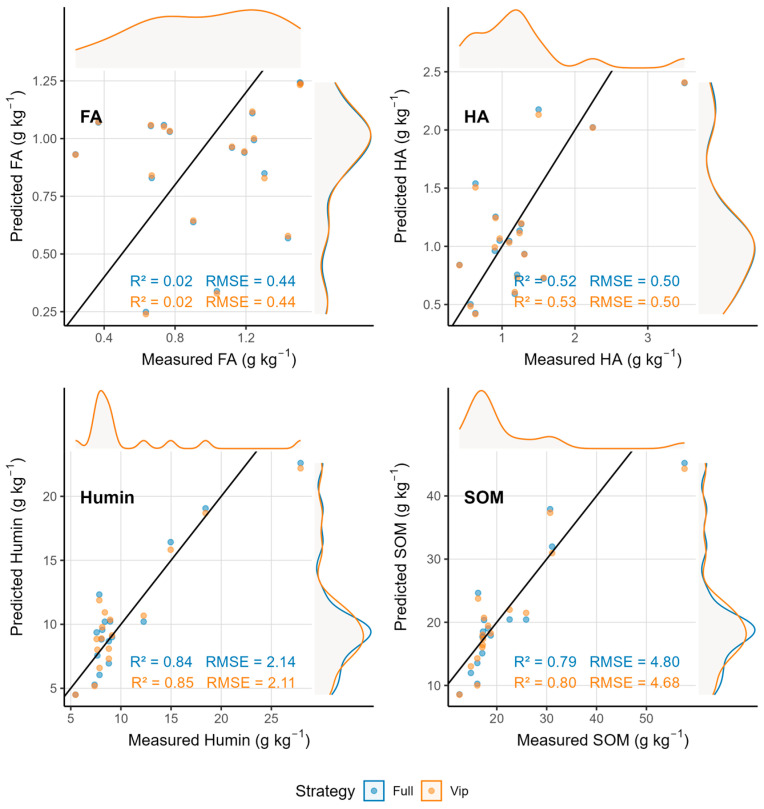
Scatter plots of measured vs. predicted values using vis–NIR spectroscopy.

Partial least squares regression models calibrated using vis–NIR spectroscopy demonstrated variable prediction accuracy across SOM fractions (Table 3, Figure 4 and Figure 5). Soil organic matter achieved strong predictive performance with validation R^2^ values of 0.79 (full spectrum) and 0.80 (VIP bands), RMSE of 4.98–5.01 g kg^−1^, and CCC values of 0.88–0.89, indicating excellent agreement between measured and predicted values. Humin exhibited comparable or superior performance, with validation R^2^ values of 0.84 (full spectrum) and 0.84 (VIP bands), RMSE of 3.36–3.38 g kg^−1^, and CCC values of 0.91 for both approaches. The use of LASSO-selected wavelengths slightly improved SOM and HA predictions by reducing model complexity and minimizing overfitting, as evidenced by decreased RMSE and bias relative to full-spectrum models.

Humic acid showed moderate predictability with validation R^2^ values of 0.37–0.38, RMSE of 1.58–1.61 g.kg^−1^, and CCC of 0.55–0.60. Scatter plots revealed that predictions for high HA concentrations exhibited substantial dispersion, whereas low-concentration predictions clustered more closely along the 1:1 line. In stark contrast, fulvic acid demonstrated very poor predictability with validation R^2^ of approximately 0.24, RMSE of 0.71 g kg^−1^, and CCC of 0.47, reflecting weak spectral expression and high prediction uncertainty. Scatter plots for FA showed wide dispersion around the 1:1 line with no clear linear trend, confirming the inability of vis–NIR spectroscopy to reliably quantify this fraction. Regression coefficient profiles (Figure 4) revealed that SOM and Humin models displayed larger coefficient amplitudes across multiple wavelength regions, particularly in the 1400–1900 nm range associated with organic C–H and O–H overtones, whereas FA and HA exhibited smaller and smoother coefficient variations.

### 3.4. MIR Prediction Accuracy for SOM and Its Fractions

**Figure 6:**
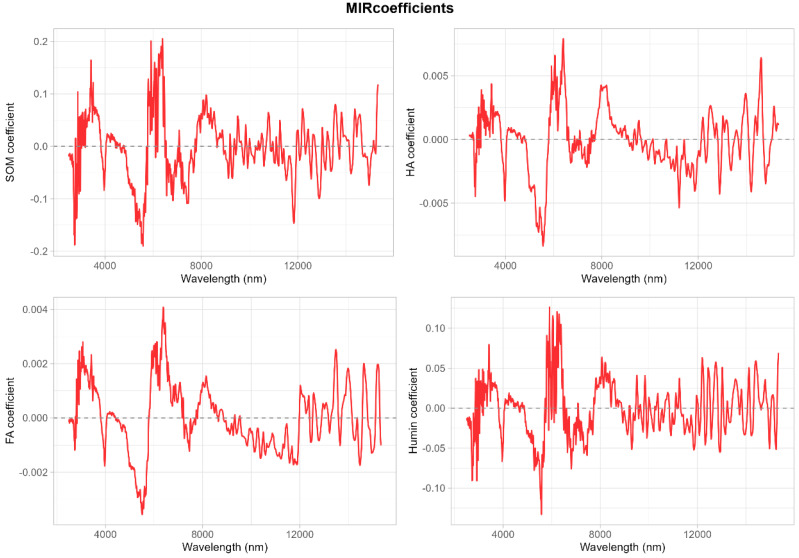
Regression coefficients for SOM and its fractions using the full MIR spectra.

**Figure 7:**
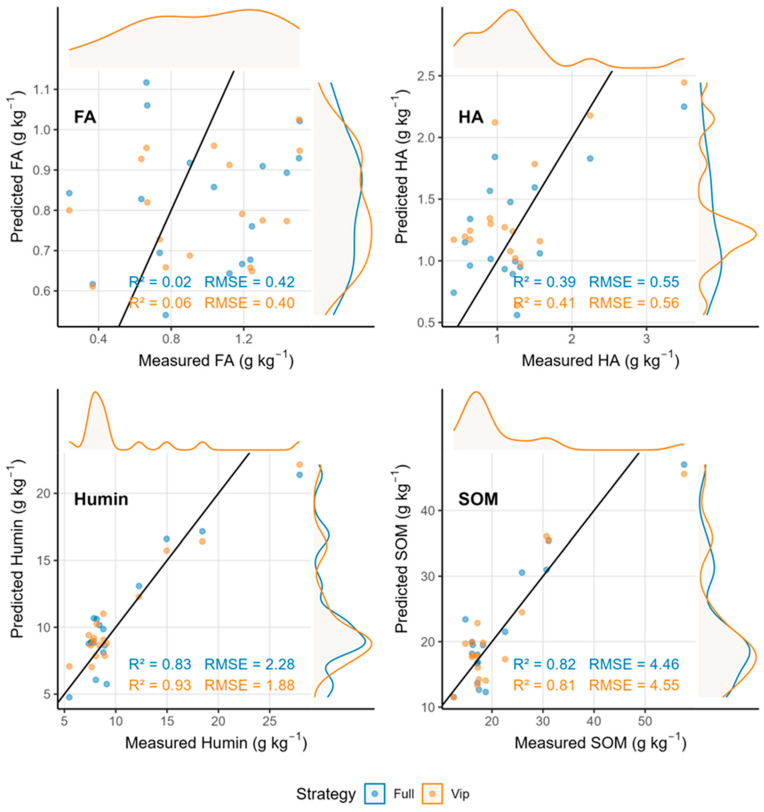
Scatter plots of measured vs. predicted values using MIR spectroscopy.

Mid-infrared spectroscopy yielded prediction accuracies comparable to or exceeding those of vis–NIR for SOM and Humin (Table 4; Figure 6 and Figure 7). Soil organic matter validation R^2^ values reached 0.82 (full spectrum) and 0.90 (VIP bands), with RMSE of 3.87–4.37 g kg^−1^ and CCC values of 0.85–0.95, demonstrating excellent predictive capability. Humin achieved the highest validation R^2^ of 0.93 (VIP bands), with RMSE of 2.19 g kg^−1^ and CCC of 0.95, representing outstanding model performance. The VIP-based MIR models for both SOM and Humin substantially outperformed their full-spectrum counterparts, indicating that wavelength selection effectively removed redundant and noisy spectral regions while retaining critical absorption features associated with organic functional groups.

Humic acid exhibited moderate MIR predictability with validation R^2^ of 0.38–0.39, RMSE of 1.58–1.66 g kg^−1^, and CCC of 0.57–0.62, similar to vis–NIR performance. Fulvic acid remained poorly predicted by MIR, with validation R^2^ of 0.15–0.23, RMSE of 0.71–0.75 g kg^−1^, and CCC of 0.37–0.47. Scatter plots confirmed that MIR-based FA predictions showed extensive dispersion without discernible linear relationships, reinforcing the conclusion that FA lacks strong, distinctive MIR absorption features. Regression coefficient analysis (Figure 6) revealed that SOM and Humin models displayed pronounced coefficient amplitudes in the 3000–8000 nm region (2.5–1.25 μm^−1^), corresponding to fundamental C–H, C=C, and C=O stretching vibrations characteristic of organic matter. In contrast, HA and particularly FA showed smaller and more diffuse coefficient profiles with maximum absolute values below 0.06, reflecting weak spectral signatures.

### 3.5. Spectral Fusion Does Not Improve Prediction Accuracy

**Figure 8:**
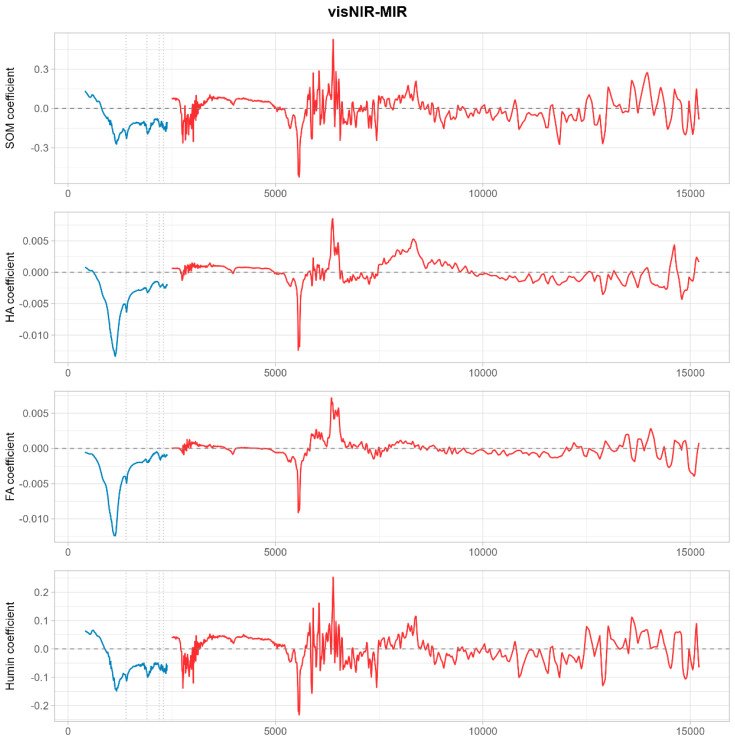
Regression coefficients for SOM and its fractions using fused spectra.

**Figure 9:**
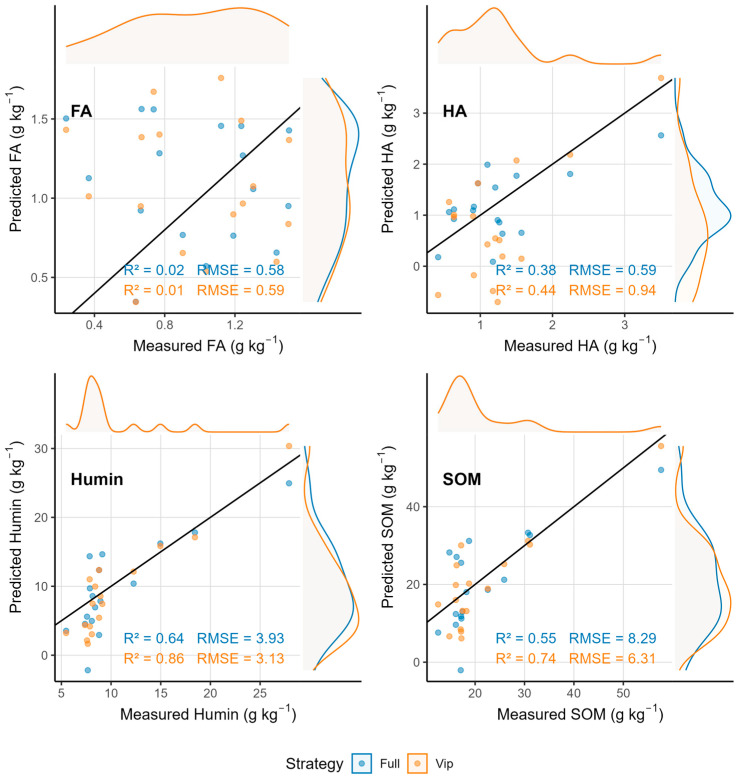
Scatter plots of measured vs. predicted values using fused spectroscopy.

Contrary to expectations, direct fusion of vis–NIR and MIR spectra did not enhance prediction accuracy for SOM or its fractions relative to the best-performing single-domain models (Table 5; Figure 8 and Figure 9). For SOM, the VIP-based fusion model achieved a validation R^2^ of 0.74, which was lower than that of the MIR-only VIP model (R^2^ = 0.90). The full-spectrum fusion model performed even more poorly (R^2^ = 0.55), suggesting that direct concatenation introduced scale imbalance and redundancy without increasing the effective information dimensionality. Humin fusion models exhibited a validation R^2^ of 0.80–0.89, which were also inferior to that of the MIR-only VIP model (R^2^ = 0.93). Humic acid and fulvic acid showed minimal or no improvement from fusion, with FA remaining unpredictable regardless of the fusion strategy.

Analysis of wavelength selection patterns after fusion revealed a reduction in the total number of selected features compared to the sum of individual region models (Table 6). For SOM, informative wavelengths decreased from approximately 41–53 across separate vis–NIR and MIR models to 18–22 after fusion. Similarly, Humin wavelengths reduced from 45–57 to 20–25. This reduction indicates that many wavelengths from both spectral regions provided overlapping information and that fusion did not introduce new, independent spectral features. Scatter plots of fusion model predictions (Figure 9) exhibited broader dispersion around the 1:1 line compared to single-region models, particularly for SOM and HA, confirming decreased prediction precision after fusion.

### 3.6. Spectral Redundancy Analysis Using Singular Value Decomposition

**Figure 10:**
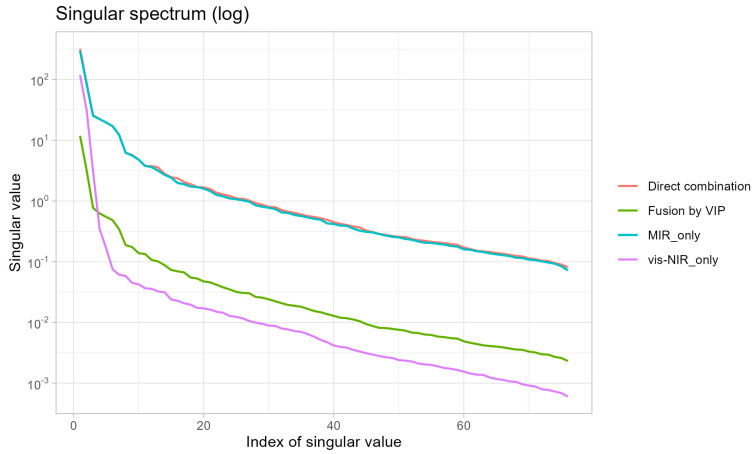
Singular value decay curves for spectroscopy before and after fusion.

Singular value decomposition analysis provided mechanistic insights into why spectral fusion failed to improve predictions (Figure 10; Table 6). The singular value decay curves revealed that the vis–NIR spectra exhibited the most rapid singular value decay, with nearly all variance captured by the first few components, indicating strong internal collinearity. The mid-infrared spectra showed intermediate decay rates, suggesting moderate spectral redundancy. The fused vis–NIR–MIR spectrum displayed a decay pattern dominated by MIR characteristics due to the larger amplitude and greater number of MIR wavelengths, which effectively overwhelmed the vis–NIR signal. This scale imbalance meant that direct concatenation did not substantially increase the effective information dimensionality but instead introduced redundancy and magnified noise from the less informative spectral region.

Cross-validation results before and after fusion (Table 6) demonstrated that the majority of selected wavelengths in the fused models originated from the MIR region. For SOM, approximately 80 percent of the top 50 selected wavelengths came from MIR, with only 20 percent from vis–NIR. This wavelength distribution confirms that MIR provided the dominant spectral information and that vis–NIR contributed minimal additional predictive value in the fusion context. The failure of fusion to enhance predictions therefore reflects the already comprehensive information content of individual spectral regions, particularly MIR, rather than inadequacies in fusion methodology.

## 4. Discussion

### 4.1. Humin Fraction Determines SOM Spectral Predictability

This study provides compelling evidence that the spectroscopic predictability of total SOM is fundamentally determined by the Humin fraction rather than by humic or fulvic acids. Both vis–NIR and MIR spectroscopy achieved high prediction accuracy for SOM and Humin (R^2^ = 0.79–0.93, CCC = 0.85–0.95); the prediction accuracy achieved in this study for SOM is broadly comparable to the best-performing results reported in the past decade of soil spectroscopy research (Table 7), whereas FA remained unpredictable (R^2^ < 0.24) and HA showed only moderate predictability (R^2^ = 0.37–0.39). This differential predictability arises from the distinct chemical and spectroscopic properties of these fractions. Humin contains abundant and spectrally active functional groups, including aromatic and aliphatic C–H, C=O, and C=C moieties, which produce strong and characteristic absorption features in both the vis–NIR and MIR regions [29]. The large number of characteristic wavelengths selected for Humin (Figure 3) and the high regression coefficient amplitudes (Figure 4, Figure 6 and Figure 8) confirm its strong spectral signature, indicating a stronger and more coherent spectral signal for Humin and helping to explain its superior predictability.

Equally important, Humin constitutes approximately 40–69 percent of total SOM in the study samples (Table 2), making it the dominant fraction by mass. This high proportional abundance means that variations in Humin content directly translate into variations in total SOM and that the strong spectral predictability of Humin is effectively transferred to bulk SOM. The structural stability of Humin, arising from strong associations with Fe/Al oxides and clay minerals [30], further contributes to its consistent spectral behavior across samples. These findings strongly support the conclusion that accurate quantitative estimation of SOM depends primarily on the predictability and relative contribution of the Humin fraction [31], rather than on the summed contributions of all fractions.

### 4.2. Chemical Characteristics Explain Fulvic Acid Unpredictability

The poor spectroscopic predictability of fulvic acid observed in this study can be attributed to its unique chemical and structural characteristics. Fulvic acid exhibits low molecular weight, high polarity, and a diffuse distribution of functional groups, resulting in weak and broad infrared absorption bands that are easily masked by overlapping signals from water, clay minerals, and other soil constituents [32]. The small number of characteristic wavelengths selected for FA (Figure 3) and the low regression coefficient amplitudes (Figure 4, Figure 6 and Figure 8) provide empirical evidence for its weak spectral signature. Moreover, FA represents a relatively small (mean approximately 3 g kg^−1^) and chemically unstable fraction within total SOM (Table 2), further diminishing its spectral detectability and statistical predictability.

Comparative studies support this interpretation. Although both HA and FA contain aliphatic and aromatic structures, HA is more chemically condensed, exhibits stronger absorption features, and demonstrates greater structural stability [32]. Fulvic acid, by contrast, shows weaker absorption, more dispersed functional groups, and lower resistance to microbial decomposition [9]. These intrinsic chemical differences fundamentally limit the capacity of vis–NIR and MIR spectroscopy to quantify FA, regardless of the modeling approach or wavelength selection strategy. Alternative analytical techniques, such as fluorescence spectroscopy or nuclear magnetic resonance, may be more suitable for characterizing FA due to their sensitivity to specific structural and electronic properties.

**Table 7:** Summary of representative SOM/SOC spectroscopy fusion studies.

Research	Study Area	Soil Type	Spectral Range	Model(s)	SOC/SOM R^2^	Fractions Included
[15]	China	Paddy and upland soils	vis–NIR + MIR	PLSR, SVM	0.78–0.90	No
[23]	China	Red soils, paddy soils	vis–NIR + MIR	RF, Cubist	0.60–0.80	No
[24]	Australia	Mixed agricultural soils	vis–NIR + MIR	PLSR, SVM	0.70–0.85	No
[14]	China	Paddy soils	vis–NIR + MIR	PLSR	0.85–0.86	No
[21]	China	Red, black, calcareous soils	MIR	PLSR	0.75–0.88	No
[33]	Germany	Loess, Cambisols	vis–NIR + MIR	PLSR	0.60–0.75	No
[26]	Brazil	Oxisols, Ferralsols	vis–NIR + MIR	Ensemble, Stacking	0.80–0.90	No
[22]	China	Mixed farmland soils	vis–NIR	CNN, PLSR	0.78–0.86	No
[27]	Egypt	Arid sandy/clay soils	vis–NIR + MIR	PLSR, RF, SVR	~0.85	No

### 4.3. Spectral Fusion Introduces Redundancy Without Information Gain

The failure of vis–NIR–MIR fusion to enhance prediction accuracy in this study contrasts with some previous reports but aligns with theoretical expectations when individual spectral regions already contain comprehensive information. Singular value decomposition analysis (Figure 10) revealed that direct concatenation of vis–NIR and MIR spectra did not substantially increase the effective information dimensionality. Instead, fusion introduced spectral redundancy because both regions captured overlapping compositional information, albeit through different physical mechanisms (electronic transitions in vis–NIR versus molecular vibrations in MIR). The reduction in the total number of selected wavelengths after fusion compared to separate regional models (Table 6) confirms this redundancy.

Additionally, direct concatenation created scale imbalance between spectral regions due to differences in measurement units (reflectance versus absorbance), wavelength density, and signal amplitude. The dominance of MIR-derived wavelengths in fused models (approximately 80 percent of selected features from MIR; Table 6) indicates that the MIR signal effectively overwhelmed the vis–NIR contribution, preventing complementary information integration. This scale imbalance can introduce noise and degrade model performance, particularly when one spectral region is already sufficiently informative. Advanced fusion strategies, such as hierarchical modeling, weighted concatenation, or deep learning architectures that learn optimal region-specific transformations, may overcome these limitations by explicitly addressing scale and redundancy issues [13]. However, for the soil samples and target properties examined in this study, single-domain spectroscopy (particularly MIR with wavelength selection) provided optimal prediction accuracy with reduced model complexity.

### 4.4. Implications for Soil Carbon Monitoring

The findings of this study have important implications for spectroscopic soil carbon monitoring programs and SOM research. First, the strong predictability of SOM and Humin using either vis–NIR or MIR spectroscopy confirms the viability of rapid, non-destructive SOM quantification across diverse agricultural and environmental applications. Mid-infrared spectroscopy with LASSO-based wavelength selection emerged as the most accurate approach (validation R^2^ up to 0.93 for Humin), suggesting that portable MIR sensors could enable cost-effective field-scale SOM assessment. Second, the component-specific predictability patterns identified here underscore the need to shift from bulk SOM estimation toward fraction-specific modeling approaches that explicitly account for the compositional heterogeneity and differential spectral behaviors of SOM pools.

Third, the central role of the Humin fraction in determining SOM predictability suggests that future spectroscopic studies should prioritize mechanistic investigations of Humin-specific spectral features and their relationships to long-term carbon stabilization processes. Understanding which functional groups and mineral associations within Humin generate the strongest spectral signals could inform targeted wavelength selection and improve model interpretability. Finally, the limited benefits of spectral fusion observed here indicate that resources may be better allocated toward optimizing single-domain spectroscopy, expanding spectral libraries, and developing robust calibration transfer methods rather than pursuing multi-sensor fusion for routine SOM monitoring applications.

### 4.5. Study Limitations and Future Directions

Several limitations should be acknowledged. The moderate sample size (n = 93) and geographic restriction to subtropical croplands in southeastern China limit the generalizability of the findings to other soil types, climatic zones, and land use systems. Validation with larger, more diverse datasets encompassing contrasting soil parent materials, textures, and management histories is essential to confirm whether the Humin-driven predictability of SOM represents a universal phenomenon or is context-specific. Additionally, the operational fractionation scheme employed (alkaline extraction and acid fractionation) provides chemically defined pools that may not directly correspond to ecologically meaningful SOM fractions defined by turnover rates or functional roles. Complementary studies integrating spectroscopy with advanced characterization techniques such as solid-state nuclear magnetic resonance, pyrolysis–gas chromatography–mass spectrometry, or synchrotron-based X-ray spectroscopy could elucidate the specific molecular structures responsible for strong Humin spectral signatures. Future research should also explore alternative fusion strategies, including hierarchical ensemble modeling and deep learning architectures, to determine whether more sophisticated integration methods can overcome the redundancy and scale imbalance limitations identified in this study. In addition, comprehensive uncertainty quantification will be addressed in future work.

## 5. Conclusions

This study systematically evaluated the capability of visible–near-infrared, mid-infrared, and fused spectroscopy for predicting soil organic matter and its chemically defined fractions. The major conclusions are as follows. First, both vis–NIR and MIR spectroscopy were able to predict soil organic matter and the Humin fraction, with MIR showing the best quantitative agreement with reference measurements (R^2^ up to 0.93); fulvic acid cannot be reliably quantified by any spectroscopic approach due to weak spectral signatures arising from low molecular weight, diffuse functional group distribution, and small fractional abundance. Second, the reliable predictability of total SOM derives primarily from the strong spectral response and high proportional contribution of the Humin fraction, which comprises approximately 50 percent of SOM and exhibits abundant spectrally active functional groups. Third, direct fusion of vis–NIR and MIR spectra does not enhance prediction accuracy because both spectral regions already contain comprehensive, overlapping information, and fusion introduces redundancy and scale imbalance without increasing effective dimensionality.

These findings establish that accurate spectroscopic estimation of SOM fundamentally depends on the predictability and relative abundance of the Humin fraction, providing new mechanistic understanding of soil carbon monitoring applications. Future research should validate these results across diverse soil types and climatic regions, investigate the specific molecular structures within Humin responsible for strong spectral signals, and develop component-specific calibration models that explicitly account for SOM compositional heterogeneity. For routine soil organic matter assessment, optimized single-domain spectroscopy (particularly MIR with variable selection) offers greater accuracy and simplicity than multi-sensor fusion approaches.

## Figures and Tables

**Table 1:** Overview of vis–NIR and MIR spectral fusion methods.

Fusion Level	Method	Core Idea and Modeling	Main Advantages	Limitations	Representative Studies
Data-level	Direct Concatenation	Concatenate vis–NIR and MIR spectra and model jointly (PLSR/SVR/RF/GBDT). Often requires block-scaling.	Simple; fully utilizes spectral information.	High dimensionality and redundancy; block dominance risk.	[15,23]
Feature-level	Dual-branch DL (CNN/MLP)	Feed vis–NIR and MIR into two network branches; fuse latent features for prediction.	Captures nonlinear patterns; end-to-end optimization.	Requires large datasets; limited interpretability.	[14,24]
	SO-PLS	Extract block-wise latent variables and sequentially integrate to reduce redundancy.	Preserves spectral block structure; interpretable.	Requires manual block ordering.	[21]
	Cross-domain Feature Selection (VIP/LASSO)	Select high-importance wavelengths across or after concatenation.	Effective dimensionality reduction; stable performance.	Primarily linear; may ignore interactions.	[15,25]
	Optimal Band Combination (OBC: RI/DI/NDI/PI/SI)	Enumerate vis–NIR × MIR band pairs and compute spectral indices; select top correlated pairs.	Few variables; strong physical interpretability.	Captures only first-order interactions; computationally intensive.	[15]
	Outer Product Analysis (OPA)	Construct cross-domain interaction features via tensor/outer product expansion.	Explicit cross-spectral interaction modeling.	High dimensionality; heavy computation.	[26]
Decision-level	Bayesian Model Averaging (BMA)	Combine predictions from multiple models using probability weighting.	Reduces model uncertainty; simple implementation.	Does not explicitly model feature interactions.	[22]
	Stacking Ensemble	Use predictions of base learners as inputs to a meta-learner (EN/GBM/MLP).	Strong generalization and robustness.	Requires out-of-fold prediction to avoid leakage.	[27]

**Table 2:** Descriptive statistics of SOM and its fractions in calibration and validation datasets.

Attribute (g kg^−1^)	Calibration					Validation				
	Mean	SD	Min	Max	Skew	Mean	SD	Min	Max	Skew
Soil Organic Matter (SOM)	26.21	12.95	13.29	67.02	1.31	21.57	10.68	12.52	57.64	2.71
Humic Acid (HA)	13.14	6.41	6.91	34.51	1.42	10.45	5.48	5.48	27.91	2.43
Fulvic Acid (FA)	0.95	0.62	0.03	2.84	1.02	0.97	0.39	0.24	1.51	−0.31
Humin	1.51	0.88	0.5	4.98	1.45	1.24	0.73	0.42	3.5	2.04

**Table 3:** Prediction accuracies using vis–NIR spectroscopy.

Target	ncomp	Calibration				Validation					
		RMSE	MAE	R^2^	Bias	RMSE	MAE	R^2^	RPIQ	CCC	Bias
SOM (Full)	4	5.88	4.06	0.79	0.00	4.80	3.56	0.79	1.32	0.88	(0.91)
SOM (VIp)	2	5.58	3.96	0.81	(0.00)	4.68	3.18	0.80	1.35	0.88	(0.88)
HA (Full)	3	0.51	0.41	0.66	(0.00)	0.50	0.39	0.49	0.81	0.69	(0.09)
HA (Vip)	2	0.50	0.40	0.67	0.00	0.50	0.39	0.51	0.82	0.70	(0.10)
FA (Full)	2	0.53	0.41	0.24	(0.00)	0.44	0.38	(0.36)	1.31	0.14	(0.09)
FA (Vip)	1	0.53	0.41	0.24	(0.00)	0.44	0.38	(0.37)	1.30	0.14	(0.09)
Humin (Full)	4	2.94	2.28	0.79	(0.00)	2.14	1.65	0.84	0.59	0.91	(0.05)
Humin (Vip)	2	2.80	2.16	0.81	0.00	2.11	1.60	0.84	0.60	0.91	(0.04)

RMSE, root mean square error (g kg^−1^); MAE, mean absolute error; R^2^, coefficient of determination; RPIQ, ratio of prediction to inter-quartile range; CCC, concordance correlation coefficient. The numbers in parentheses are negative.

**Table 4:** Prediction accuracies using MIR spectroscopy.

Target	Method	ncomp	Calibration			Validation					
			RMSE	MAE	R^2^	RMSE	MAE	Bias	R^2^	RPIQ	CCC
FA	Full	2	0.49	0.39	0.36	0.42	0.38	(0.15)	(0.23)	1.38	0.08
FA	VIP	2	0.49	0.38	0.35	0.41	0.35	(0.17)	(0.15)	1.42	0.12
HA	Full	3	0.51	0.38	0.67	0.55	0.47	0.00	0.39	0.74	0.56
HA	VIP	5	0.45	0.37	0.73	0.56	0.46	0.13	0.38	0.73	0.58
Humin	Full	7	1.31	1.06	0.96	2.28	1.79	0.04	0.83	0.56	0.89
**Humin**	**VIP**	**7**	**1.59**	**1.20**	**0.94**	**1.88**	**1.34**	**0.16**	**0.88**	**0.68**	**0.92**
SOM	Full	6	2.68	1.95	0.96	4.46	3.37	0.05	0.81	1.42	0.90
SOM	VIP	7	2.94	2.11	0.95	4.55	3.66	(0.18)	0.81	1.39	0.89

**Table 5:** Prediction accuracies using fused vis–NIR and MIR spectroscopy.

Target	Strategy	ncomp	Calibration			Validation					
			R^2^	RMSE	MAE	R^2^	RMSE	MAE	Bias	RPIQ	CCC
SOM	Full	7	0.78	6.41	4.43	0.55	8.29	6.84	(1.05)	0.76	0.73
SOM	VIP	5	0.88	4.44	3.44	0.74	6.31	4.90	(1.42)	1.00	0.84
HA	Full	2	0.35	0.72	0.54	0.38	0.59	0.52	(0.07)	0.69	0.61
HA	VIP	5	0.59	0.57	0.44	0.39	0.94	0.69	(0.39)	0.43	0.52
FA	Full	2	0.23	0.54	0.40	0.02	0.58	0.47	0.12	1.00	(0.12)
FA	VIP	2	0.40	0.48	0.36	0.00	0.59	0.48	0.01	1.00	(0.01)
Humin	Full	7	0.76	3.38	2.33	0.64	3.93	3.10	(0.85)	0.32	0.78
Humin	VIP	8	0.94	1.62	1.28	0.89	3.13	2.47	(1.29)	0.41	0.89

**Table 6:** Cross-validation results and wavelength distribution after fusion.

Target	Cross-Validation Results Before and After Fusion				The Number of Top VIP		
	RMSE	RMSE_sd	Fused_RMSE	Fused_RMSE_sd	TopK	From_vis	From_mir
SOM	7.80	0.66	7.09	0.73	50	37	13
HA	0.78	0.03	0.74	0.03	50	28	22
FA	0.63	0.02	0.55	0.02	50	37	13
Humin	4.34	0.35	3.98	0.60	50	34	16

## Data Availability

The authors have permission to share only a limited portion of the data used in this study.
